# Supplementary material for: How minimizing conflicts could lead to polarization on social media: An agent-based model investigation
Source: PLoS One. 2022 Jan 27;17(1):e0263184. doi: 10.1371/journal.pone.0263184 (PMC8794152; doi:10.1371/journal.pone.0263184)
Supplement: S1 File — All the supporting figures. (PDF) [file pone.0263184.s001.pdf]

Supplementary Information for "How Minimizing Conflicts Could Lead to Polarization on Social Media: an Agent-Based Model Investigation"

Michele Coscia[1*], Luca Rossi[1],

1 IT University of Copenhagen, Rued Langgaards Vej 7, Copenhagen, Denmark

* mcos@itu.dk

1 Normal Polarity Distribution in Real World Data

One of the starting conditions of our ABM is that users and sources polarities follow a normal distribution. This implies that that most users are moderates; more extreme users/sources are progressively more rare, at both ends of the spectrum.

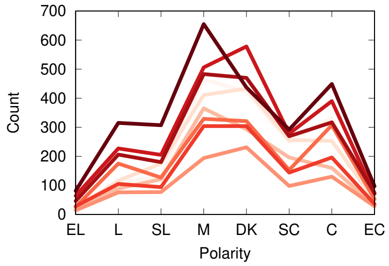

Fig 1. The polarity distribution in the US from 1994 (light) to 2016 (dark). Biannual observation, except for missing years 2006, 2010, and 2014. EL = Extremely Liberal, L = Liberal, SL = Slightly Liberal, M = Moderate, DK = Don't Know, SC = Slightly Conservative, C = Conservative, EC = Extremely Conservative.

This assumption is supported by real world data. Figure 1 shows the distribution of political leaning in the USA across time [1][1]. These data were collected by surveying a representative sample of the US electorate via phone and face-to-face interviews.

While not perfectly normally distributed, the data show that the majority of Americans either feel they are moderate or do not know to which side they lean. "Moderate" or "don't know" is always the mode of the distribution, and their combination is always the plurality option.

2 Parameter Ranges

In the main paper we claim that the parameter range we focus on is the one supporting most of the variation of the system. Here we provide supporting evidence for this claim, examining the range of each parameter in turn (except σ, for which we already explore the wide 0.1-1 range).

[1]https://electionstudies.org/resources/anes-guide/top-tables/?id=29

2.1 Reshareability (ρ)

The parameter ρ is the one for which we explore the narrowest space. There are two reasons for this choice: one theoretical and one practical.

The theoretical reason is that ρ's value is capped by ϕ. A world with $\rho \geq \phi$ is unreasonable, because it would be a scenario where a user feels enough indignation by an item that they will flag it, but then they will also reshare it to their social network. Thus, we only test scenarios in which $\rho < \phi$. Since the lowest value of ϕ we test is 0.1, that is also the maximum ρ value we can test.

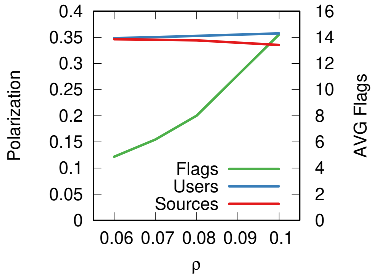

Fig 2. The evolution of polarization for users (in blue) and sources (in red) and the total number of flags (in green) per value of ρ (x axis), across all runs and other parameter combinations.

The practical reason can be appreciated by examining Figure 2. Here we see that ρ has a limited impact on polarization – something we already concluded in Section 4.1 of the main paper. The only thing ρ affects is the number of flags in the system – because higher ρ means that the users will share news items at greater distances from their opinion.

Thus, Figure 2 is telling us that increasing ρ means that users and sources will arrive in the same place – because their polarization lines are flat – but faster – because there are more flags injected in the system.

2.2 Tolerance (ϕ)

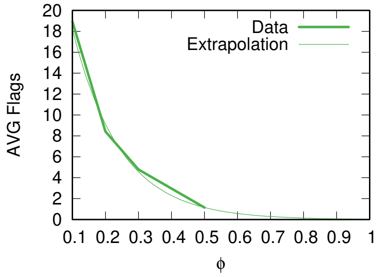

Fig 3. The number of flags (y axis) in the model for different values of ϕ (x axis), summing across all runs and other parameter combinations. Thick line = data from experiments; thin line: extrapolation.

In the paper, the maximum value of ϕ we consider is 0.5. The motivation can be found in Figure 3. The figure shows the average number of flags per source generated per value of ϕ. The higher the ϕ, the fewer the flags, as the user finds more news items acceptable. The trend shows that, for $\phi > 0.5$, we do not have a sufficient number of flags to support our observation of the model's behaviour – sources receive less than one flag on average.

2.3 Integrity (γ)

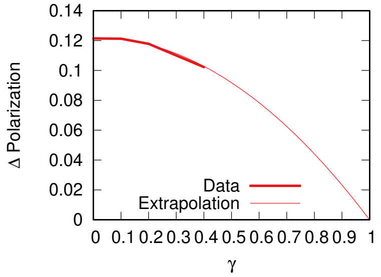

Fig 4. The average difference in polarization between initial and final condition (y axis), for increasing values of integrity γ (x axis). Thick line = data from experiments; thin line: extrapolation.

In the paper, the maximum value of γ we consider is 0.4. High γ create static simulations that do not change much because the news sources have a high integrity and do not move their polarization, no matter the inputs they receive. Figure 4 shows that indeed the average polarization movement for sources decreases as γ increases. The slope gets more prominent as γ increases – and the change is 0 for $\gamma = 1$, by definition.

3 Convergence

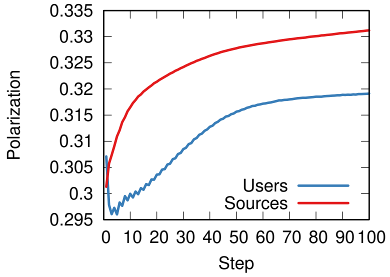

Fig 5. The average source and user polarization (y axis) as the model evolves over time (x axis).

The ABM we present in the paper is too complex for us to be able to prove and guarantee its convergence. However, we can analyze its behavior as the simulation progresses step by step and record its salient characteristics. In Figure 5 we track the average user and source polarization across all parameter values per time step. We can see that, after around 40 steps for sources and 50 steps for users, both sources and users have found a stable polarization regime, whose rate of change tends to go down as the simulation proceeds.

4 Robustness

One simplifying assumption made in our original model is that sources only react to flagging, by trying to move their polarity so that it would minimize the amount of backlash they receive. Sources, however, are more complex than this: they also want to maximize their audiences. Thus, they might want to accept a polarity change if that comes with an increased audience, even if there are a few more flags.

Would our results significantly change if we were to make this addition? To study this question, we modify the role of the γ parameter – Section 3.4.2 in the main paper. Rather than testing it against $F_{s,t-1}$ – which is a normalized decrease in number of flags received –, we test it against a linear combination of $F_{s,t-1}$ and $S_{s,t-1}$.

$S_{s,t-1}$ is built like $F_{s,t-1}$, but actually measures relative audience increase:

$$S_{s,t} = \frac{|\overline{A_{s,t}}| - |A_{s,t}|}{|A_{s,t}| + 1},$$

with $|\overline{A_{s,t}}|$ being the size of the audience of source s at time t if s were to move its polarity.

How much s should weight $F_{s,t-1}$ and $S_{s,t-1}$ should be regulated by yet another parameter. However, for the purpose of this robustness test, we assume that s will weigh them equally.

To estimate the effect of this change in the results of our model, we calculate the Wasserstein distance between the polarity distribution of the regular model with the one we obtain with this variant. We sample some of the 12,800 combinations of parameter values and runs. We obtain an average distance of 0.039 and 0.062 for the polarity distributions of users and sources, respectively. Note that the maximum value for this metric is 2, since that is the distance between a vector of -1s and a vector of 1s.

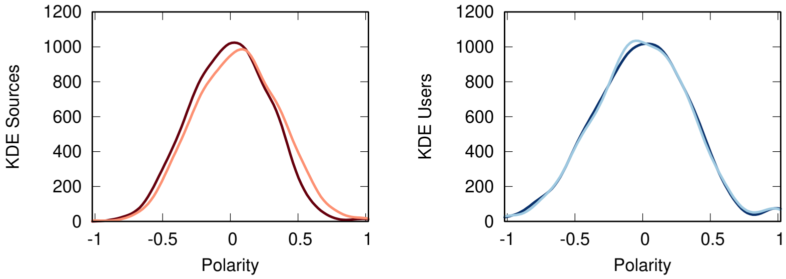

Fig 6. The comparison in the polarity distributions of sources (left, red) and users (right, blue) for the median case. Regular model in bright color, variant in dark color. The plot shows the KDE estimate of sources and users (y axis) at a given polarity level (x axis).

To help put these values into context, we pick a representative (median) run and we plot the polarity distributions in Figure 6. In the median run, the distance between sources is 0.056. We can see from the figure that the model variant tends to moderate sources and to pull them towards neutrality, but the actual difference is small.

This is the main reason why we do not include this factor in the main model, as the impact of this mechanic does not justify the added complexity of introducing a fifth parameter.

5 Sample Run

To aid intuition about the model's behavior, we provide a run through one instance of one parameter combination. We decide to set the parameters as follows: $\rho = 0.1$, $\phi = 0.1$, $\gamma = 0.2$, and $\sigma = 1.0$. We choose these parameters because they are the ones better reproducing the abortion dataset we obtained from Twitter – as we show in Table 2 in the main paper.

We start showing the evolution of the distributions of source and user polarity – in Figure 7. Note that the distributions here look more noisy than the ones in the paper,

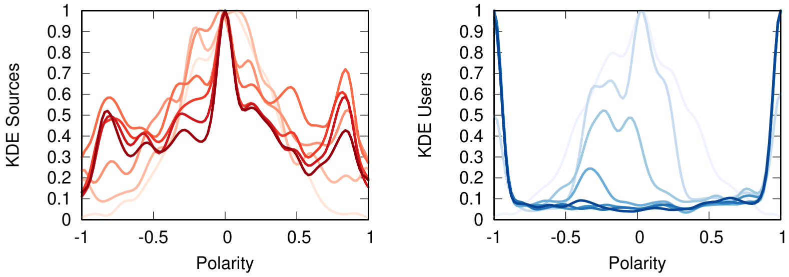

Fig 7. Kernel density estimations (y axis) of sources (red, left) and users (blue, right) at a given polarity value (x axis). The simulation runs from brighter to darker colors. Here we show the distributions at steps (from bright to dark) 0, 16, 32, 48, 64, 80, 96.

because we are showing a single run, while the distributions in the paper are the aggregate over all 50 runs made with a given parameter configuration.

The polarity distributions show how sources move chaotically until three main peaks emerge: neutral and polarized on either side of the spectrum. On the other hand, users quickly move to the extremes. This is in line with the real world user polarity distribution. We show it in 8(left): barred an x-axis normalization factor, the real world data shows two strong peaks at either side of neutrality with little in between, just like the output of the model.

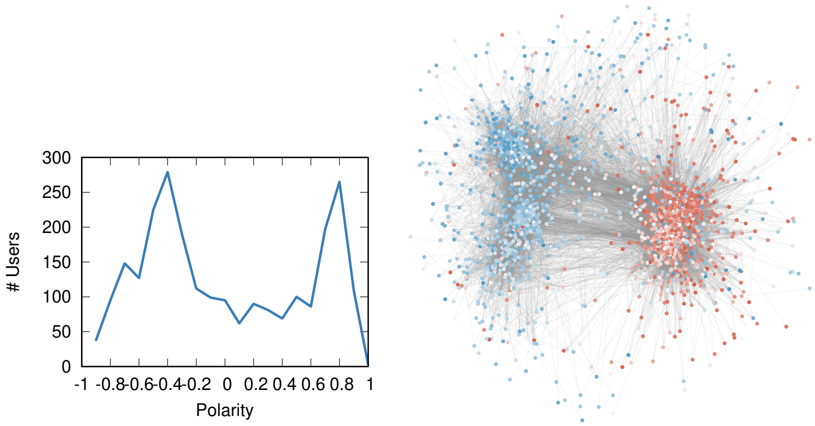

Fig 8. (Left) The user polarity distribution in the abortion network on Twitter. (Right) The abortion network on Twitter, with users connected if they follow each other and colored according to their polarity value.

We now look at how the social network among users evolve. We show the same steps we used for the distribution of polarity values, except the first step. To see the initial condition, you can use Figure 2b in the main paper. Figure 9 shows how it evolves from step 16 to step 96.

From the figure we can see that the users organize on a polarity spectrum early on: the starting community structure is already gone by step 16. The rest of the evolution shows the emergence of two communities of extremists, while neutral users either drop out of the network or are attracted to extreme polarity values.

Note how the model is able to correctly create the two communities of extremists, which are also a prominent topological feature of the real world Twitter network (Figure 8(right)).

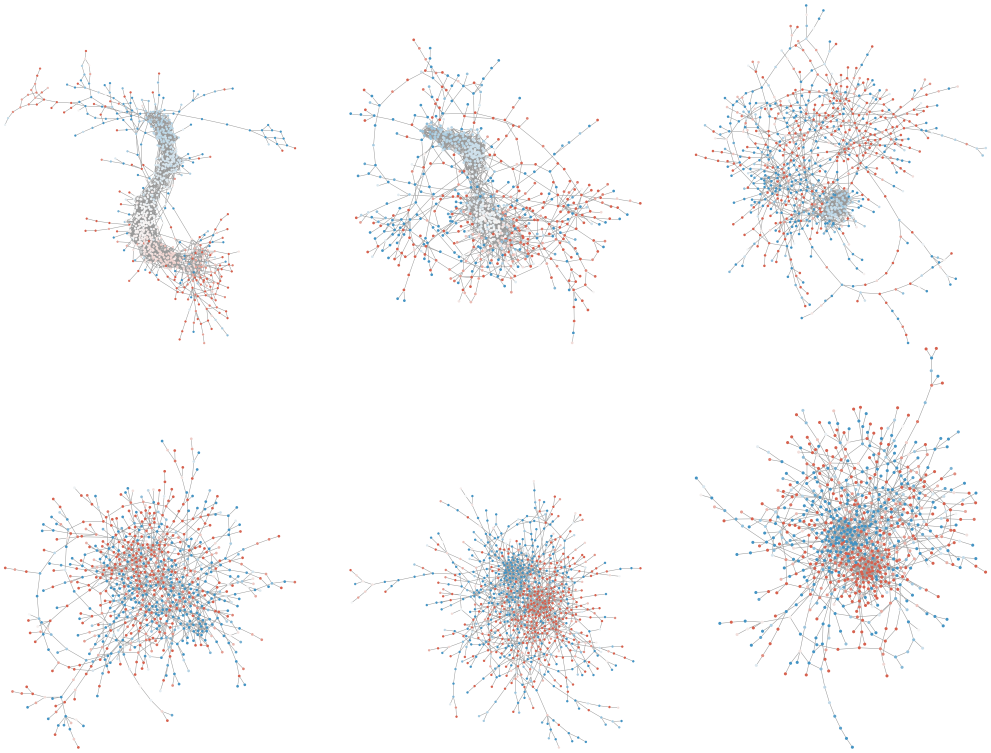

Fig 9. The social network in the sample run in steps (top row, left) 16, 32, 48, and (bottom row, left to right) 64, 80, 96.

References

1. Studies ANE. The ANES guide to public opinion and electoral behavior; 2010.
