# Supplementary material for: How minimizing conflicts could lead to polarization on social media: An agent-based model investigation
Source: PLoS One. 2022 Jan 27;17(1):e0263184. doi: 10.1371/journal.pone.0263184 (PMC8794152; doi:10.1371/journal.pone.0263184)
Supplement: Attachment — Submitted filename: response_reviewers.pdf [file pone.0263184.s002.pdf]

Cover Letter for the paper “How Minimizing Conflicts Could Lead to Polarization on Social Media: an Agent-Based Model Investigation”

Michele Coscia & Luca Rossi
IT University of Copenhagen

December 14, 2021

To the editors of PLOS One,

we are writing this letter to accompany our paper titled “How Minimizing Conflicts Could Lead to Polarization on Social Media: an Agent-Based Model Investigation”. We have addressed and/or responded to all comments raised by the reviewers and we find the paper much improved because of that. Please find our point-by-point reply to the reviewers below. All edits in the paper are also available in a track-changes edited version, to make it easier to follow our changes.

We hope that you and the reviewers will agree with us about the improvements of the paper.

We look forward to hearing from you.

Yours faithfully,
Michele Coscia & Luca Rossi
IT University of Copenhagen
mcos@itu.dk, lucr@itu.dk

Reviewer #1

1. The authors claim they can reasonably reproduce the results for a dataset from the reference I suggested and used Wasserstein distance to confirm this result. However, I have still hold doubts regarding the users' distributions. Figure S7 shows the distribution obtained by the model for the parameters that best fit Twitter data. However, the distribution is quite different from the marginal one shown in figure 1a of the reference I mentioned. Indeed figure S7 of this paper show a high peak at the center that is not present in the top marginal distribution figure 1a of the suggested reference.

- Part of the reason why Fig S7 did not look like Fig 1a in the reference was that we worked with the "guncontrol" data and the reference worked with the "abortion" data. We have now switched to using the "abortion" data to make the comparison possible. However, the main reason for the difference rested on the fact that our validation relied on an unmet assumption. Specifically, we assumed that mediabiasfactcheck allowed sources to take polarity values in the full $-1/+1$ polarity spectrum. This is not true: no source in mediabiasfactcheck has a polarity of either -1 or $+1$, making those polarities unattainable for users. Thus, this makes a direct comparison with our model imperfect, because our ABM allows users to take values in the full $-1/+1$ polarity spectrum – which is routinely done in the literature (e.g. Wang et al, Physical Review X, 2020). We have now normalized the mediabiasfactcheck data to have the same value domain as our model, under the assumption that we are aligning our theoretical maximum/minimum values with the observed maximum/minimum values of polarization. We also used a more intuitive test relying on the Pearson correlation coefficient in Sections 4 and S5. As can be seen comparing Fig S7(right) with the real world distribution (Fig S8 (left)), the two distributions exhibit similar features, with two large peaks at either side of neutrality and little in between, if Fig S8's x axis were to be normalized. Moreover, even the topology of the model's network (Fig S9 (bottom right)) shows the main two-community feature of the real world data (Fig S8 (right)).

2. Nevertheless, the authors did not show the distribution of users polarization obtained by the Twitter data they retrieved, and thus I cannot say whether or not their model can effectively reproduce it in case it has significant differences from the one shown in the reference. (...) Hence, I suggest the authors show the real data distribution.

   - We now show the real world data in Fig S8, both polarity distribution and network topology.

3. I think the paper is missing a brief discussion on the parameters that best fit real data to help the reader understand which characteristics we need to fit Twitter data (e.g. high/low sharing, tolerance, etc.)

   - We have now added this as the conclusion of Section 4. Specifically we show that high sharing, low tolerance, and high volatility reproduce well the real world data, while integrity seems not to be playing a role.

Reviewer #2

1. I've noticed that the authors still missed two important references (which were also mentioned in my previous report) that addressed the coupling

evolutions and the polarization of both users and news outlets using agent-based models, and I suggest the authors include them in the introductory discussion (...)

- We apologize for this oversight and we have now included these two papers in our introductory discussion. They are now Ref 6 (Wang et al) and Ref 10 (Schmidt et al).
